# Variabilidade da Frequência Cardíaca como Indicador de Risco Cardiovascular em Jovens

**DOI:** 10.36660/abc.20200444

**Published:** 2020-07-28

**Authors:** Breno Quintella Farah

**Affiliations:** 1 Departamento de Educação Física Universidade Federal Rural de Pernambuco RecifePE Brasil Departamento de Educação Física da Universidade Federal Rural de Pernambuco (UFRPE), Recife , PE - Brasil; 2 Programa de Pós-Graduação em Educação Física UFPE RecifePE Brasil Programa de Pós-Graduação em Educação Física da UFPE Recife , PE - Brasil

**Keywords:** Hipertensão, Pressão Arterial, Hereditariedade/genética, Futebol, Atletas, Esportes para Jovens, Endotélio/função

O sistema nervoso autônomo, que é composto pelos sistemas simpático e parassimpático, desempenha papel importante na regulação das funções de diversos sistemas do corpo humano, como o sistema cardiovascular. O controle neural do coração está diretamente ligado às alterações na frequência cardíaca e à atividade reflexa barorreceptora, cuja oscilação decorre de estímulos ambientais. ^1 , 2^ Tais estímulos podem levar à redução da frequência cardíaca por meio do sistema nervoso parassimpático, o qual atua na diminuição da frequência de despolarização do nódulo sinusal pela ação da acetilcolina na junção neuroefetora cardíaca. Por sua vez, o sistema nervoso simpático promove uma elevação da frequência cardíaca por meio da liberação de noradrenalina, que age via ligação com os receptores β-adrenérgicos, aumentando o ritmo de despolarização do marca-passo sinusal. ^1 , 2^

Em indivíduos saudáveis ou atletas, com sistema nervoso autônomo íntegro, observa-se predominância da modulação parassimpática em detrimento da modulação simpática para o coração. Por outro lado, em indivíduos com doenças cardiovasculares, como a hipertensão arterial, esse padrão está invertido, com maior modulação simpática e menor modulação parassimpática sendo observadas, o que caracteriza um quadro de disfunção autonômica cardíaca. ^1 - 3^ Isso é particularmente importante, visto que alterações na modulação autonômica cardíaca e, consequentemente, na frequência cardíaca podem repercutir diretamente no débito cardíaco, de modo a repercutir em alterações na pressão arterial (PA). ^1^

Especificamente na população jovem, estudos também têm demonstrado associação entre disfunção autonômica cardíaca e aumento da PA em diversos estudos. Em estudo prévio do nosso grupo, ^4^ observamos, em uma amostra de 1.152 adolescentes homens (14 a 19 anos de idade), que níveis elevados de PA estão diretamente associados a maior modulação simpática e menor modulação parassimpática cardíaca, independentemente do nível de atividade física e estado nutricional que também afetam a modulação autonômica cardíaca. ^5 - 7^

O estudo “História familiar de hipertensão prejudica o balanço autonômico, mas não a função endotelial em jovens jogadores de futebol” ^8^ buscou comparar a modulação autonômica, a função endotelial e o consumo máximo de oxigênio (VO _2max_ ) de jovens atletas (saudáveis), separados de acordo com a história de PA dos seus pais, a fim de investigar a influência da ascendência genética nesses parâmetros. Para tanto, foi conduzido um estudo transversal, no qual 46 jogadores de futebol (18±2 anos) foram separados de acordo com a PA dos seus pais: 1) pai e mãe normotensos; 2) somente pai hipertenso; 3) somente mãe hipertensa; e, 4) pai e mãe hipertensos.

Um ponto de destaque do estudo é o nível controlado de PA dos atletas, bem como a excelente condição de saúde. Nesse sentido, nenhuma diferença foi encontrada entre os atletas na função endotelial e no VO _2max_ . Por outro lado, os autores encontraram que os atletas cujos pais são hipertensos apresentaram disfunção autonômica, enquanto os atletas com pais normotensos apresentavam sistema autonômico cardíaco íntegro. A novidade do estudo citado é a sugestão de que, antes do comprometimento da PA, comum em filhos de hipertensos, ^9^ há a disfunção autonômica com manutenção da função endotelial mesmo em jovens atletas com bom nível de saúde. Previamente, já havia sido demonstrado, na população em geral, que filhos de hipertensos apresentam disfunção autonômica cardíaca, ^10^ mas não havia sido demonstrada a relação com jovens atletas saudáveis.

Esses resultados sugerem que a avaliação da modulação autonômica cardíaca pode ser usada na avaliação do risco cardiovascular já em jovens, a fim de adotar condutas preventivas. Dentre as diversas formas de avaliar a modulação autonômica cardíaca, destaca-se a variabilidade da frequência cardíaca (VFC), por ser um método simples e não invasivo de avaliação do sistema nervoso autonômico com base nas oscilações dos intervalos entre batimentos cardíacos consecutivos (intervalos R-R) ^11^ com boa reprodutibilidade intraindivíduos e inter e intra-avaliador. ^12^ Para análise da VFC, os parâmetros podem ser obtidos por meio de métodos lineares, no domínio do tempo e da frequência, e métodos não lineares. ^11^

Nesse contexto, dada a quantidade numerosa de parâmetros que podem ser avaliados na VFC e a ausência de pontos de cortes universalmente aceitos, o uso da VFC na prática clínica ainda é incipiente, mesmo que, em 1996, uma força-tarefa da European Society of Cardiology (ESC) e da North American Society of Pacing and Electrophysiology (NASPE) ^11^ buscou padronizar e estabelecer o uso clínico dos parâmetros da VFC. Especificamente com adolescentes, nosso grupo4 e outros ^13 , 14^ descreveram valores de referência em amostras representativas da população em geral de adolescentes, bem como o estabelecimento de ponto de corte para os parâmetros lineares da VFC na identificação de risco cardiovascular, ^15^ facilitando assim o uso na prática clínica.

Desse modo, fica evidente a importância da avaliação da modulação autonômica cardíaca, dada a sua relação com fatores de risco cardiovasculares até mesmo em amostra saudável. O estudo “História familiar de hipertensão prejudica o balanço autonômico, mas não a função endotelial em jovens jogadores de futebol”8 reforça esse achado. No entanto, sendo um estudo transversal com amostra pequena, obviamente, apresenta limitações. Assim, futuros estudos podem considerar ampliar essa investigação aumentando o número de atletas na amostra, inclusive do sexo feminino.
