# Supplementary material for: Variabilidade da Frequência Cardíaca como Indicador de Risco Cardiovascular em Jovens
Source: Arq Bras Cardiol. 2020 Jul 28;115(1):59–60. [Article in Portuguese] doi: 10.36660/abc.20200444 (PMC8384311; doi:10.36660/abc.20200444)
Supplement: Translation

# Translation: Heart Rate Variability as an Indicator of Cardiovascular Risk in Young Individuals

**Authors:** Breno Quintella Farah

**Affiliations:** 1 Brazil Departamento de Educação Física da Universidade Federal Rural de Pernambuco (UFRPE), Recife, PE - Brazil; 2 Brazil Programa de Pós-Graduação em Educação Física da UFPE, Recife, PE - Brazil

**Keywords:** Hypertension, Blood pressure, Heredity / genetics, Soccer, Athletes, Youth Sports, Endothelial / function

The autonomic nervous system, which consists of the sympathetic and parasympathetic systems, plays an important role in regulating the functions of several systems in the human body, such as the cardiovascular system. Neural control of the heart is directly related to changes in heart rate and baroreceptor reflex activity, of which oscillation results from environmental stimuli.^1 , 2^ These stimuli can lead to heart rate reduction through the parasympathetic nervous system, by decreasing the frequency of sinus node depolarization through the effect of acetylcholine on the cardiac neuroeffector junction. In turn, the sympathetic nervous system promotes an increase in heart rate through the norepinephrine release, which acts through the connection with β-adrenergic receptors, increasing the depolarization rate of the sinus pacemaker.^1 , 2^

Healthy individuals or athletes, with an intact autonomic nervous system, show a predominance of parasympathetic modulation to the detriment of sympathetic modulation to the heart. On the other hand, in individuals with cardiovascular diseases, such as arterial hypertension, this pattern is reversed, with greater sympathetic modulation and less parasympathetic modulation being observed, which characterizes a picture of cardiac autonomic dysfunction.^1 - 3^ This is particularly important, considering that changes in cardiac autonomic modulation and, consequently, in heart rate, can have a direct impact on cardiac output, which can result in BP changes.^1^

Specifically in the young population, several studies have also shown an association between cardiac autonomic dysfunction and increased BP. In a previous study by our group,^4^ we observed, in a sample of 1152 male adolescents (aged 14 to 19 years), that high BP levels are directly associated with greater sympathetic modulation and less cardiac parasympathetic modulation, irrespective of the level of physical activity and the nutritional status, which also affect cardiac autonomic modulation.^5 - 7^

The study “Family History of Hypertension Impairs the Autonomic Balance, but not the Endothelial Function, in Young Soccer Players”^8^ sought to compare autonomic modulation, endothelial function and maximum oxygen consumption (VO_2max_) in young (healthy) athletes, divided according to their parents’ blood pressure (BP) history, aiming to investigate the influence of genetic inheritance on these parameters. For that purpose, a cross-sectional study was carried out, in which 46 soccer players (18 ± 2 years old) were divided according to their parents’ BP levels: 1) normotensive father and mother; 2) hypertensive father only; 3) hypertensive mother only; and, 4) hypertensive father and mother. One of the highlights of the study is the athletes’ BP control, as well as their excellent health status. In this sense, no difference was found among the athletes regarding endothelial function and VO_2max_. On the other hand, the authors found that athletes whose parents are hypertensive had autonomic dysfunction, while athletes with normotensive parents had an intact cardiac autonomic system.

The novelty of the aforementioned study is the suggestion that before the BP impairment, common in children of hypertensive patients,^9^ there is autonomic dysfunction with maintenance of endothelial function even in young athletes with good health status. It had been previously shown in the general population that children of hypertensive patients have cardiac autonomic dysfunction,^10^ but the association with healthy young athletes had not been demonstrated.

These results suggest that the cardiac autonomic modulation evaluation can be used to assess cardiovascular risk in young individuals, aiming at establishing preventive measures. Among the different ways of assessing cardiac autonomic modulation, heart rate variability (HRV) stands out, because it is a simple and non-invasive method of assessing the autonomic nervous system based on oscillations in the intervals between consecutive heartbeats (RR-intervals)^11^ with good intra-individual, and inter- and intra-evaluator reproducibility.^12^ For HRV analysis, the parameters can be obtained using linear methods, in the time and frequency domain, as well as non-linear methods.^11^

In this context, given the many parameters that can be evaluated in HRV and the lack of universally accepted cutoff points, the use of HRV in clinical practice is still incipient, even though, in 1996, a task force of the European Society of Cardiology and the North American Society of Pacing and Electrophysiology^11^ sought to standardize and establish the clinical use of HRV parameters. Specifically with adolescents, our group^4^ and others^13 , 14^ described reference values in representative samples of the general adolescent population, as well as the establishment of a cutoff point for the HRV linear parameters in the identification of cardiovascular risk,^15^ thus facilitating the use in clinical practice.

That said, the importance of assessing cardiac autonomic modulation is evident, given its association with cardiovascular risk factors even in a healthy sample. The study “Family History of Hypertension Impairs the Autonomic Balance, but not the Endothelial Function, in Young Soccer Players”^8^ reinforces this finding. However, being a cross-sectional study with a small sample, it obviously has some limitations. Thus, further studies may consider expanding this investigation by increasing the number of athletes in the sample, including female ones.
